# Modulation of the Structure and Stability of Novel Camel Lens Alpha-Crystallin by pH and Thermal Stress

**DOI:** 10.3390/gels8050273

**Published:** 2022-04-27

**Authors:** Ajamaluddin Malik, Javed Masood Khan, Abdullah S. Alhomida, Mohammad Shamsul Ola

**Affiliations:** 1 Department of Biochemistry, College of Science, King Saud University, Riyadh 11451, Saudi Arabia; alhomida@ksu.edu.sa (A.S.A.); mola@ksu.edu.sa (M.S.O.); 2 Department of Food Science and Nutrition, Faculty of Food and Agricultural Sciences, King Saud University, Riyadh 11451, Saudi Arabia; jmkhan@ksu.edu.sa

**Keywords:** alpha-crystallin, dynamic multimode spectroscopy, circular dichroism, fluorescence, thermal stability

## Abstract

Alpha-crystallin protein performs structural and chaperone functions in the lens and comprises alphaA and alphaB subunits at a molar ratio of 3:1. The highly complex alpha-crystallin structure challenges structural biologists because of its large dynamic quaternary structure (300–1000 kDa). Camel lens alpha-crystallin is a poorly characterized molecular chaperone, and the alphaB subunit possesses a novel extension at the N-terminal domain. We purified camel lens alpha-crystallin using size exclusion chromatography, and the purity was analyzed by gradient (4–12%) sodium dodecyl sulfate–polyacrylamide gel electrophoresis. Alpha-crystallin was equilibrated in the pH range of 1.0 to 7.5. Subsequently, thermal stress (20–94 °C) was applied to the alpha-crystallin samples, and changes in the conformation and stability were recorded by dynamic multimode spectroscopy and intrinsic and extrinsic fluorescence spectroscopic methods. Camel lens alpha-crystallin formed a random coil-like structure without losing its native-like beta-sheeted structure under two conditions: >50 °C at pH 7.5 and all temperatures at pH 2.0. The calculated enthalpy of denaturation, as determined by dynamic multimode spectroscopy at pH 7.5, 4.0, 2.0, and 1.0 revealed that alpha-crystallin never completely denatures under acidic conditions or thermal denaturation. Alpha-crystallin undergoes a single, reversible thermal transition at pH 7.5. The thermodynamic data (unfolding enthalpy and heat capacity change) and chaperone activities indicated that alpha-crystallin does not completely unfold above the thermal transition. Camels adapted to live in hot desert climates naturally exhibit the abovementioned unique features.

## 1. Introduction

Alpha-crystallin belongs to the small heat shock protein (sHsp) superfamily, is highly expressed in the eye lens, and has at least two known functions. First, alpha-crystallin is a structural protein that maintains an appropriate refractive index (ability to focus light on the retina). Second, as a molecular chaperone, it maintains lens clarity throughout the lifespan of an organism [1]. Eye lens proteins are frequently exposed to environmental stress, including UV-radiation and high temperatures. The mature lens fibers lack a protein folding machinery and all organelles to minimize light scattering. Consequently, no new protein can be synthesized, and damaged proteins cannot be replaced. Therefore, the eye lens in all organisms must maintain damaged proteins such as alpha-crystallin in a soluble state throughout life. The camel has adapted to thrive in extreme desert climates, which includes high temperatures, solar radiation, dryness, and low nutrition. High ambient temperature and UV–Vis radiation may increase the lens temperature and induce protein misfolding and aggregation [2]. Epidemiological studies have shown a positive association between early-onset and a high grade of cataracts and prolonged sunlight exposure [3,4]. Therefore, the role of the camel lens in maintaining lenticular alpha-crystallin in a soluble state throughout its entire life presents challenges. Unfolding and aggregation of lenticular proteins cause lens opacity, resulting in cataract formation.

Lens alpha-crystallin is a large, heterogeneous multimeric protein comprising two subunits (alphaA and alphaB chains), each approximately 175 amino acids and exhibiting 60% homology. In the human eye lens, alpha-crystallin comprises 15–50 subunits of two homologous forms, alphaA and alphaB, each approximately 20 kDa [5,6] and at a 3:1 molar ratio [5]. Camels have evolved uniquely (anatomically, physiologically, and biochemically) to adapt to the scorching climate where most other mammals cannot survive. To our best knowledge, the camel eye lens has at least two novel features: the recruitment of high levels of taxon-specific zeta-crystallin and the presence of an extended N-terminal domain in the alphaB-crystallin protein. Camel alphaA-crystallin (XP_010998042.1) comprises 173 amino acid residues, identical to human alphaA-crystallin. However, camel alphaB-crystallin (XP_010984284.1) contains an additional 44 residues compared with human alphaB-crystallin (219 residues vs. 175 residues, respectively) [7].

The expression of alphaA-crystallin is primarily lens specific; in other tissues, it is expressed in trace amounts. By contrast, alphaB-crystallin expression is stress-inducible and widespread throughout the body, particularly in the heart, muscle, and brain [8,9]. AlphaB-crystallin overexpression is linked to several protein misfolding and neurodegenerative diseases, including myopathies [10,11], Parkinson’s disease [12,13], Alzheimer’s disease [14,15], Creutzfeldt–Jakob disease [16,17], multiple sclerosis [18,19], and cancer [20,21].

Alpha-crystallin acts as a “holdase” in an ATP-independent manner [1,22]. The size of the hetero-oligomeric quaternary structure of alpha-crystallin is diverse with an average molecular weight of 700 kDa, and its size ranges from 300 to 1000 kDa [5,23]. The size variation is caused by several factors (e.g., pH, ionic strength, temperature, and metal ions). Temperature is a critical factor for alpha-crystallin oligomerization [23,24]. Recently, we reported that the chaperone activity of camel alpha-crystallin is activated in a stepwise manner during heat stress [7]. Moreover, camel alpha-crystallin retains a native beta-sheeted dominant secondary structure up to 50 °C. High thermal stress (above 50 °C) leads to a structural transition in alpha-crystallin with a gain of a random-coiled-like structure without losing beta-sheeted content [7]. In previous studies of alpha-crystallin, temperature was associated with the single thermal transition and activation of its chaperone activity [25,26].

Thermal transition was reported to occur above 50 °C with a transition mid-point (*T*_m_) of approximately 61–63 °C [7,27,28,29]. Interestingly, alpha-crystallin efficiently retained its function during and above thermal transition [7,30,31]. Surprisingly, the calculated enthalpy of denaturation (ΔH) for alpha-crystallin at pH 7.5 using different techniques [Differential scanning calorimetry (DSC), dynamic multimode spectroscopy (DMS), and Fourier transform infrared spectroscopy (FTIR)] was significantly lower than the theoretically estimated enthalpy of denaturation [7,32]. The experimentally calculated heat capacity change of alpha-crystallin denaturation (ΔCp) was also less than half of the theoretical (ΔCp) value [26,32,33]. Several reports have shown the structural integrity of alpha-crystallin under thermal denaturation at pH 7.5. However, ambiguities exist regarding the folding species of alpha-crystallin above the transition. Whether alpha-crystallin is fully unfolded, partially unfolded, or retains a native-like structure remains unclear [28,29,34,35]. Alpha-crystallin protein presents challenges for structural determination, and its crystal structure is unavailable. In particular, camel lens alpha-crystallin comprises an extended N-terminal domain and is poorly characterized. In the present study, we used multi-spectroscopic techniques to characterize the folding and thermodynamic characteristics of alpha-crystallin in the pH range of 1.0–7.5 and temperature range of 20–94 °C. Many proteins are unfolded at low pH due to loos of electrostatic interactions. Several types of forces such as ionic, hydrophobic, H-bond, and covalent interactions are responsible to maintain the structure–function relationship of the proteins. The change in medium may perturb these interactions and result in protein unfolding. In this study, we have evaluated the role of pH and temperature on the stability of alpha-crystallin.

## 2. Materials and Methods

Superdex 200 and Superdex 75 prepacked columns were obtained from GE Healthcare Life Sciences, Chicago, USA. The 4–12% gradient SDS-PAGE gels were purchased from Invitrogen and Bradford’s reagent was obtained from Pierce. All other chemicals were of analytical grade.

### 2.1. Extraction and Purification of Alpha-Crystallin from Camel Lens

Fresh camel eye lenses were obtained from a local slaughterhouse and transported under chilled conditions. Two lenses were gently stirred in 50 mL of extraction buffer [20 mM sodium phosphate buffer (pH 7.8) containing 0.2 mM EDTA] for 30 min to extract the soluble lens protein. The supernatant was collected after centrifugation at 13,000 rpm for 15 min. Alpha-crystallin was purified using two different size exclusion chromatography columns (Superdex 200 and Superdex 75 gel filtration columns). The Superdex 200 and Superdex 75 gel filtration columns were equilibrated with 20 mM sodium phosphate buffer (pH 7.8) containing 0.2 mM EDTA. The clear soluble lens extract was passed through a Superdex 200 column, and the purity of the eluted fractions was evaluated by 4–12% gradient sodium dodecyl sulfate–polyacrylamide gel electrophoresis. Subsequently, fractions containing relatively pure alpha-crystallin were pooled and passed through a Superdex 75 gel filtration column. The fraction purity was re-analyzed by 4–12% sodium dodecyl sulfate–polyacrylamide gel electrophoresis [7]. The pure fractions were concentrated to 8 mg/mL and stored at −20 °C. The protein was quantified using the Bradford assay.

### 2.2. Equilibration of Alpha-Crystallin at Different pH Values

Camel lens alpha-crystallin (0.3 mg mL^−1^) was equilibrated overnight with a 20 mM buffer (pH 1.0–7.5) at room temperature. To obtain the desired pH, the following buffers were used: KCl-HCl (pH 1.0 and 1.5), Gly-HCl (pH 2.0–3.0), acetate buffer (pH 4.0–5.0), and phosphate buffer (pH 6.0–7.5).

### 2.3. Far-UV CD Spectroscopy of Alpha-Crystallin at Different pH Values

Far-UV CD measurements of alpha-crystallin equilibrated at different pH values were obtained using a Chirascan^Plus^ spectropolarimeter (Applied Photophysics Ltd., London, UK) and coupled with a Peltier temperature controller. The far-UV CD spectra of alpha-crystallin were measured at a concentration of 0.3 mg/mL in a 1-mm-pathlength cuvette at 22 °C. Three spectra for each sample were scanned from 200 to 250 nm with a 1-nm bandwidth, and the data were collected at 0.5 s per point. The air baseline and buffer background were subtracted from each spectrum of alpha-crystallin.

### 2.4. Intrinsic Fluorescence Spectroscopy of Alpha-Crystallin at Different pH Values

The tryptophan fluorescence emission spectra of alpha-crystallin at different pH values were measured at room temperature using a Cary Eclipse Fluorescence Spectrophotometer (Agilent Technologies, Santa Clara, CA, USA) coupled with a Peltier temperature controller [36]. Alpha-crystallin (0.1 mg/mL) at different pH values (1.0 to 7.5) in a 10-mm-pathlength cuvette was excited at 295 nm (bandwidth, 5 nm each) to record tryptophan fluorescence emission spectra.

### 2.5. Dynamic Multimode Spectroscopy of Alpha-Crystallin at Different pH Values

DMS was performed using a Chirascan^Plus^ spectrophotometer [37]. Based on the observation of major secondary and tertiary structural transitions in camel lens alpha-crystallin with respect to pH values, four different pH values (1.0, 2.0, 4.0, and 7.5) were selected for detailed spectroscopic and thermodynamic studies. Camel lens alpha-crystallin (0.2 mg/mL) was dissolved in 20 mM buffer at pH 1.0, 2.0, 4.0, and 7.5, and temperature-dependent conformational changes were measured in 1-mm-pathlength cuvettes using internal temperature probes. Alpha-crystallin was gradually heated from 20 °C to 94 °C at a rate of 1 °C/min, and far-UV CD spectra were recorded between 200 and 250 nm. The thermal transition data were processed using Chirascan Global 3 software provided by the manufacturer.

### 2.6. ANS (8-Anilino-1-naphthalene sulfonate) Fluorescence Measurements of Alpha-Crystallin at Different Temperatures and pH Values

ANS fluorescence of alpha-crystallin (0.2 mg mL^−1^) at pH 1.0, 2.0, 4.0, and 7.5, respectively, were recorded at different temperatures (5 °C increments at each step) from 20 °C to 90 °C using a Peltier-controlled Cary Eclipse Fluorescence Spectrophotometer. ANS (50 μM) was added to the alpha-crystallin samples at pH 1.0, 2.0, 4.0, and 7.5, respectively, and the samples were equilibrated for 1 h at room temperature. The solution temperature was monitored using an internal temperature probe. ANS-treated alpha-crystallin was gradually heated and allowed to equilibrate for 5 min at each temperature step. The ANS fluorescence emission spectra were recorded between 400 and 600 nm (5.0-nm slit) by exciting the samples at 375 nm (2.5-nm slit).

## 3. Results

### 3.1. Effect of pH on the Secondary Structure of Camel Lens Alpha-Crystallin

**Figure 1:**
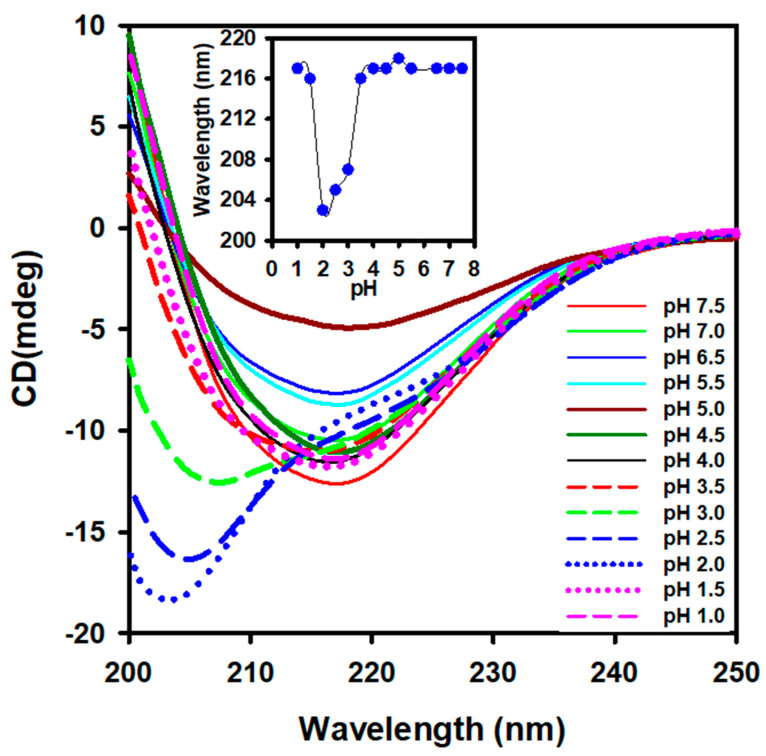
Far-UV circular dichroism (CD) spectra of 0.3 mg mL^−1^ of alpha-crystallin at pH 1.0 to 7.5. Changes in the ellipticity minima at different pH values are plotted in the inset figure. Below pH 3.5, alpha-crystallin gained a random coil-like structure but was restored to a native-like structure at pH 1.0.

Pure alpha-crystallin was obtained as previously described [7]. Far-UV CD (200–250 nm) was used to characterize the effect of acidic pH on the secondary structure of alpha-crystallin (0.3 mg mL^−1^) (Figure 1). The far-UV CD spectra of alpha-crystallin at pH 7.5 revealed a single negative minimum at 217, which is a characteristic feature of beta-sheeted proteins. Changes in the negative minima of alpha-crystallin were insignificant, between pH 4.0 and 7.5 (Figure 1, inset), but the ellipticity at 217 nm gradually decreased as the pH was reduced from 7.5 to 5.0, indicating a loss of secondary structure (beta-sheeted structure) (Supplementary Figure S1). The maximum loss of secondary structure was observed at pH 5.0. The alpha-crystallin quickly regained a beta-sheeted structure below pH 5.0, particularly at pH 4.5, 4.0, and 3.0, respectively, which was close to the native secondary structure. Interestingly, at pH 3.0, 2.5, and 2.0, respectively, the alpha-crystallin secondary structure transformed into a random coil structure without affecting its beta-sheeted core structure (Figure 1). When alpha-crystallin was further incubated at pH 1.0 and 1.5, negative ellipticity was regained to that of native alpha-crystallin (Figure 1, inset). The far-UV CD data indicated that the secondary structure of alpha-crystallin was unstable with respect to pH changes. Figure 1 shows the different folding states of alpha-crystallin in which the far-UV CD minima varied with pH: native state (pH 7.5), beginning of the random-coil-like structure (pH 4.0), random-coiled structure (pH 2.0), and gain of the native-like secondary structure (pH 1.0).

### 3.2. Effect of pH on the Tertiary Structure of Alpha-Crystallin

**Figure 2:**
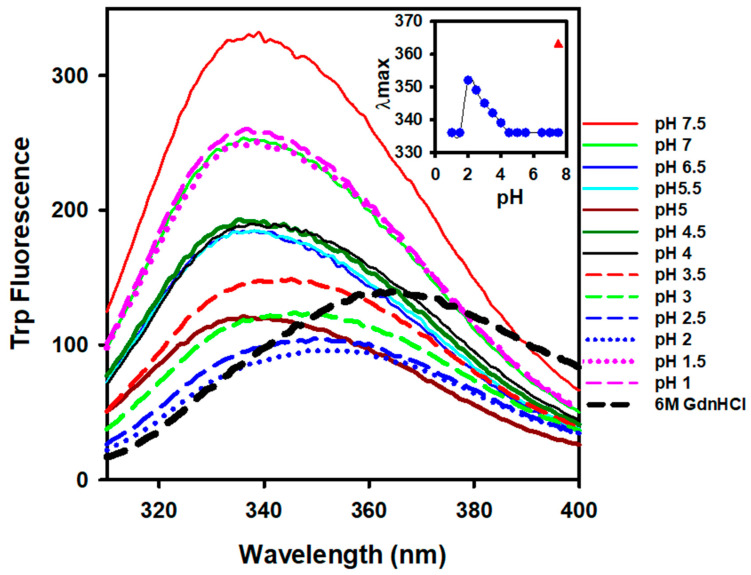
Tryptophan fluorescence spectra of camel lens alpha-crystallin at pH 1.0–7.5. Alpha-crystallin (0.1 mg mL^−1^) equilibrated at different pH values was excited at 295 nm. Emission spectra were collected from 300 to 400 nm at room temperature (bandwidth 5 nm each). Each spectrum recorded at a different pH is color-coded. The inset figure shows the λmax plot with respect to pH.

Intrinsic fluorescence spectroscopy was used to investigate changes in the alpha-crystallin tertiary structure with respect to pH changes. Measurements of intrinsic fluorescence are useful readouts of the microenvironment surrounding aromatic residues and provide information regarding even subtle changes in the tertiary structure of proteins [38,39,40]. Figure 2 shows the tryptophan fluorescence spectra of alpha-crystallin at pH 1.0 to 7.5, revealing that alpha-crystallin at pH 7.5 exhibited a maximum fluorescence intensity at 336 nm. This finding confirmed that alpha-crystallin existed in a well-folded form. As the pH was reduced, the fluorescence emission maximum (λ_max_) of alpha-crystallin was unchanged up to pH 5.5. However, below pH 5.5 down to pH 2.0, a gradual redshift in the λ_max_ was observed, indicating exposure of tryptophan residues to the polar environment (Figure 2, inset). The redshift in the wavelength maximum occurs when the microenvironment surrounding tryptophan residues becomes polar (aqueous), indicating protein unfolding or a loss of protein tertiary structure. The maximum redshift of alpha-crystallin was found at pH 2.0, indicating that the alpha-crystallin tertiary structure was maximally lost. Interestingly, at pH values below 2.0, the λ_max_ of the alpha-crystallin returned to 336 nm (i.e., the native-like structure), indicating that alpha-crystallin was again refolded at pH 2.0. The λ_max_ of alpha-crystallin was 352 nm; in the completely unfolded state (in 6 M GdnHCl), alpha-crystallin showed a λ_max_ of 363 nm, indicating a partially unfolded state of alpha-crystallin at pH 2.0.

### 3.3. Changes in Surface Hydrophobicity at Selected pH Values

**Figure 3:**
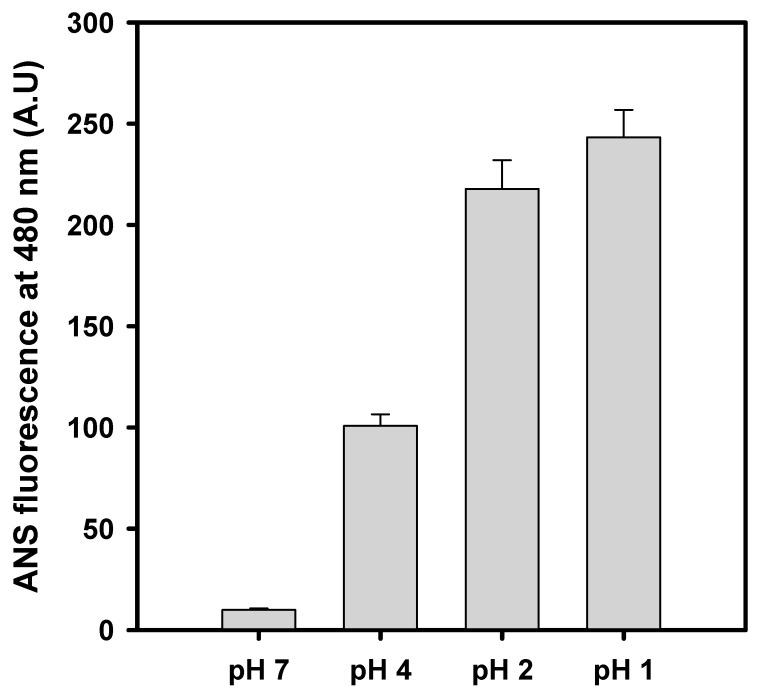
Surface hydrophobicity measurements of alpha-crystallin at four selected pH values. Alpha-crystallins (0.2 mg mL^−1^) equilibrated at pH 1.0, 2.0, 4.0, and 7.5, respectively, were treated with 50 μM ANS. The samples were excited at 375 nm (2.5 nm slit) and the emission spectra were recorded between 400 and 600 nm (5.0 nm slit).

The partially unfolded states or molten globule states of proteins are frequently characterized by measuring changes in ANS fluorescence. ANS has a significantly lower binding affinity with native and fully denatured proteins because the appropriate hydrophobic patches are unavailable for ANS binding. However, the partially unfolded or molten globule state of the protein exposes hydrophobic patches and provides a suitable environment for ANS binding and producing high ANS fluorescence intensity [41,42]. The exposure of hydrophobicity of alpha-crystallin (0.2 mg mL^−1^) at four different pH values (1.0, 2.0, 4.0, and 7.5, respectively) was measured at room temperature (Figure 3). Alpha-crystallin in the native state (pH 7.5) exhibited a poor ANS binding signal, indicating that alpha-crystallin in the native state has low surface hydrophobicity (Figure 3). This finding indicates that alpha-crystallin is well-folded. However, at pH 4.0, 2.0, and 1.0, respectively, the fluorescence intensity of ANS increased in response to a change in pH, confirming that the surface hydrophobicity of alpha-crystallin was increased with respect to the change in pH. The increased surface hydrophobicity with respect to pH resulted from the formation of the molten, globule-like state of alpha-crystallin.

### 3.4. Thermodynamic and Spectroscopic Properties of Alpha-Crystallin at Different pH Values as Determined by Dynamic Multimode Spectroscopy

**Figure 4:**
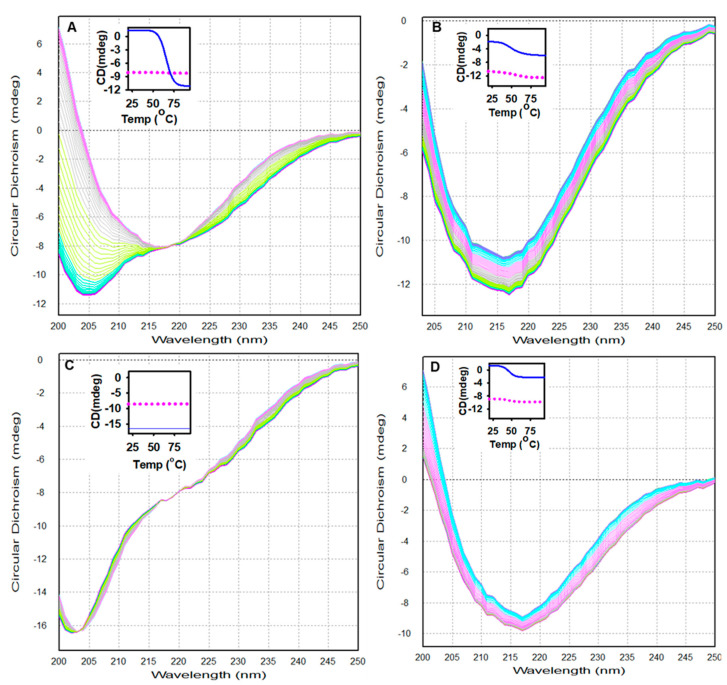
Far-UV circular dichroism (CD) spectra of camel lens alpha-crystallin at different temperatures and pH values. Alpha-crystallin (0.2 mg mL^−1^) was heat-denatured at a constant rate (1 °C min^−1^) at pH 7.5 (**A**), pH 4.0 (**B**), pH 2.0 (**C**), and pH 1.0 (**D**). Far-UV CD spectra were collected from 200 to 250 nm at intervals of 1 °C from 20 °C to 94 °C. In the inset figure, the blue line shows the changes at 203 nm and the pink dots at 217 nm, with respect to temperature.

The spectroscopic and thermodynamic properties of alpha-crystallin at four different pH values (1.0, 2.0, 4.0, and 7.5) were examined by DMS [43]. Alpha-crystallin revealed distinct secondary and tertiary structures at pH 1.0, 2.0, 4.0, and 7.5, respectively. Therefore, we selected these pH values to evaluate the thermodynamic and folding characteristics of alpha-crystallin. Alpha-crystallin samples (0.2 mg mL^−1^) at pH 1.0, 2.0, 4.0, and 7.5, respectively, were heat-stressed from 20 °C to 94 °C at a rate of 1 °C/min under identical conditions. The far-UV CD spectra (200 to 250 nm) were recorded as a function of temperature. Figure 4A–D shows the changes in the secondary structure conformation of alpha-crystallin at different temperatures and pH values. Alpha-crystallin in the native state (pH 7.5 and room temperature) exhibited a single minimum at 217 nm, representing a characteristic feature of a beta-sheeted rich protein (Figure 4A). The peaks at 217 nm were unchanged during heat stress (20–94 °C), indicating that the alpha-crystallin beta-sheeted core structure was preserved during heat stress. Moreover, the ellipticity at 217 nm was unchanged between 20 °C and 50 °C, indicating that the secondary structure was intact over this temperature range. Interestingly, above 50 °C, a sharp increase in the ellipticity minima at 203 nm was observed without altering the 217 nm ellipticity, indicating the formation of a random coil-like structure while maintaining the original beta-sheeted structure (Figure 4A, inset).

Our results also demonstrated that far-UV CD ellipticity at 217 nm was unchanged at pH 7.5 and 2.0 (Figure 4A,C). By contrast, a slight increase in the ellipticity minima at 217 nm at pH 4.0 and 1.0 (Figure 4B,D) was detected during thermal denaturation. These results indicated that the core beta-sheeted structure of alpha-crystallin remained intact during thermal denaturation (Figure 4). Moreover, the far-UV CD spectra of thermally stressed (>80 °C) alpha-crystallin at pH 7.5 were similar to those of alpha-crystallin at pH 2.0 at all temperatures (20 °C–94 °C). These data showed the presence of random-coiled and beta-sheeted structures under two conditions: alpha-crystallin above 50 °C at pH 7.5 and alpha-crystallin at all temperatures at pH 2.0 (Figure 4A,C). The far-UV CD spectra of alpha-crystallin at pH 4.0 and 1.0 were similar to native-like alpha-crystallin, and these conformations did not undergo any major structural transitions during heat stress, except a slight gain of ellipticity minima at 217 nm (Figure 4B,D). Moreover, thermal stress at pH 1.0, 2.0, 4.0, and 7.5, respectively was reversible, and no aggregation was detected.

**Figure 5:**
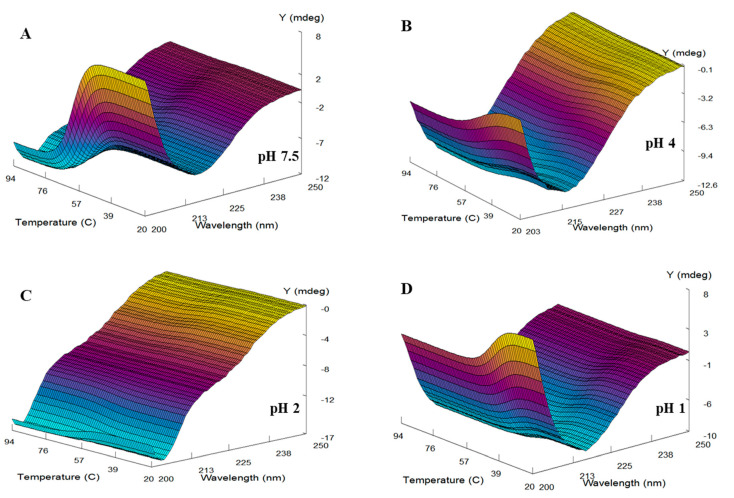
Calculated temperature, wavelength, and far-UV CD signal of alpha-crystallin at different pHs. The three-dimensional model of alpha-crystallin at pH 7.5 (**A**), 4.0 **(B**), 2.0 (**C**), and 1.0 (**D**), respectively, was calculated using Global 3 software and the far-UV CD signal obtained during temperature ramping (1 °C min^−1^).

The thermal transition midpoints (*T*_m_) and enthalpy of alpha-crystallin at pH 1.0, 2.0, 4.0, and 7.5, respectively, were determined (Table 1) using Global 3 analysis software provided by Applied Photophysics Ltd., UK. The three-dimensional model of the thermal transitions in alpha-crystallin at pH 1.0, 2.0, 4.0, and 7.5, respectively, was generated using Global 3 analysis software (Figure 5).

### 3.5. Changes in the Surface Hydrophobicity of Alpha-Crystallin at Different Temperatures and pH Values

**Figure 6:**
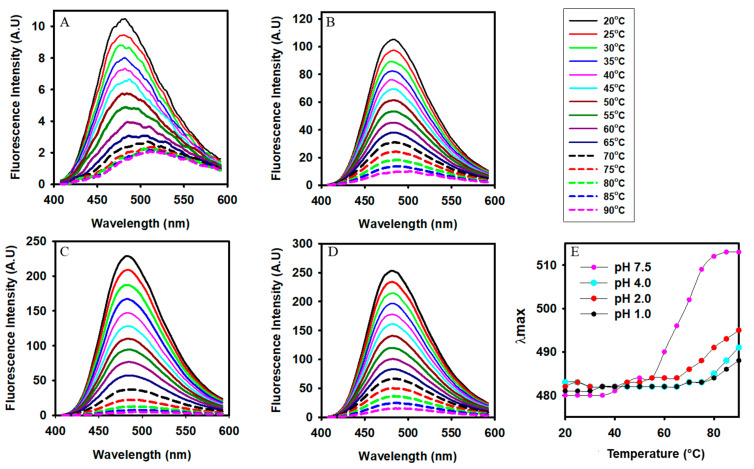
Differential scanning fluorometry of alpha-crystallin using an extrinsic fluorophore at four different pH values. ANS fluorescence was monitored by incubating alpha-crystallin at pH 7.5, 4.0, 2.0, and 1.0, respectively, from 20 °C to 90 °C by stepwise temperature increases of 5 °C. (**A**) pH 7.5, (**B**) pH 4.0, (**C**) pH 2.0, and (**D**) pH 1.0. (**E**) Effect of temperature on the λ_max_ of alpha-crystallin at pH 7.5, 4.0, 2.0, and 1.0, respectively.

Extrinsic fluorophore ANS was used to monitor the exposure of hydrophobic patches in alpha-crystallin in response to thermal stress. ANS fluorescence of alpha-crystallin at pH 1.0, 2.0, 4.0, and 7.5, respectively, was evaluated at different temperatures, from 20 °C to 90 °C (Figure 6). When alpha-crystallin at pH 7.5 was heat stressed from 20 °C, a slight redshift in the wavelength maxima was observed above 35 °C, and a sharp redshift was observed above 55 °C (Figure 6A), indicating exposure of hydrophobic residues at the surface of alpha-crystallin in response to heat stress. Alpha-crystallin at pH 7.5 exhibited low ANS fluorescence intensity and displayed poor ANS binding with native state alpha-crystallin at pH 7.5 (Figure 6A). Because ANS is a temperature-sensitive probe, a gradual decrease in ANS fluorescence intensity was observed at all pH values. At acidic pH (4.0, 2.0, and 1.0), the ANS fluorescence of alpha-crystallin exhibited increased fluorescence intensity resulting from the exposure of hydrophobic patches (Figure 6B–D). A slight redshift in emission was observed only above 65 °C when alpha-crystallin was at pH 1.0, 2.0, and 4.0, respectively (Figure 6E).

## 4. Discussion

Ocular proteins are exposed to environmental stress (solar radiation and ambient temperature), making them susceptible to unfolding and aggregation. Alpha-crystallin naturally maintains ocular proteins in a soluble state. The Arabian camel has evolved to live in a stressful desert climate of intense heat, solar radiation, and dryness. The camel possesses several unique anatomical, physiological, and biochemical features to survive and thrive in the extreme desert climate [44,45,46,47,48]. The camel eye lens has two modifications with respect to crystallin proteins: it contains levels of zeta-crystallin (a taxon-specific crystallin) [49] and an extended N-terminal domain in the alphaB-crystallin protein [7]. Camel alphaA-crystallin (NCBI Reference Sequence: XP_010998042.1) is identical in length and shares 93% homology with human alphaA-crystallin, whereas camel alphaB-crystallin (NCBI Reference Sequence: XP_010984284.1) contains 44 additional residues at its N-terminus and shows an overall 78% similarity with human alphaB-crystallin [7].

The alpha-crystallin architecture is highly complex, and its quaternary structure changes (forms larger functional oligomers) as the temperature increases. Alpha-crystallin presents challenges for structural biologists to study. Despite several attempts, the crystal structure of the alpha-crystallin has not been solved. Its three-dimensional structure has been reconstituted using multiple techniques, including cryo-electron microscopy, NMR, dynamic light scattering, analytical ultracentrifugation, and structural modeling [50,51]. Because the hetero-oligomeric quaternary structure of alpha-crystallin changes in response to temperature, interpreting the structural changes in alpha-crystallin using spectroscopic techniques precisely is challenging. We used multiple spectroscopic methods (DMS, intrinsic, and ANS fluorescence) to characterize the changes in the secondary and tertiary structure and surface hydrophobicity of camel lens alpha-crystallin in response to changes in pH and temperature.

At pH 7.5, camel lens alpha-crystallin exhibited a single minimum at 217 nm, indicating the presence of a beta-sheeted dominant structure. Earlier studies reported a beta-sheeted dominant structure in bovine alpha-crystallin at pH 7.5 [32,34]. The effect of pH from 1.0 to 7.5 on the secondary structure of camel lens alpha-crystallin revealed that the single negative minimum remained at 217 nm between pH 7.5 and 4.0. During the pH scanning experiment, multiple conformational changes were detected in the secondary structure of alpha-crystallin. Initially, loss of the beta-sheeted structure occurred up to pH 5.0. A further reduction of pH to 4.0 induced a beta-sheeted structure, resulting in the formation of native-like alpha-crystallin. A subsequent reduction of pH resulted in major conformational changes, as shown in Figure 1. Below pH 4.0, alpha-crystallin contained native-like beta-sheeted and random-coiled structures. Interestingly, at pH 1.5 and 1.0, the far-UV CD spectra nearly overlapped with alpha-crystallin at pH 7.5, indicating a restoration of the native-like beta-sheeted structure and a loss of the random-coiled structure.

For a detailed investigation of the spectroscopic and thermodynamic properties of alpha-crystallin, four different pH values were selected: pH 7.5, native alpha-crystallin; pH 4.0, native-like alpha-crystallin; pH 2.0, alpha-crystallin with random coils; and pH 1.0, native-like alpha-crystallin. DMS based on far-UV CD spectroscopy was used to obtain thermodynamic and spectroscopic data. DMS is an information-rich technique that precisely determines changes in the secondary structure of proteins under different conditions over the entire temperature range [43,52]. Figure 4A–D and Table 1 show the thermodynamic and spectroscopic data obtained by DMS. Below 50 °C (pre-transition) and at pH 7.5, alpha-crystallin exists as a beta-sheeted dominant protein (Figure 4A). It retained a native beta-sheeted secondary structure up to 50 °C at pH 7.5. However, above 50 °C, the minima shifted toward 203 nm and maintained a shoulder at 217 nm. The ellipticity at 217 nm in the pre- and post-transition spectra remained the same. Chemical denaturants (urea and single minimum) induce a decrease in ellipticity at 217 nm (loss of beta-sheeted secondary structure) and subunit dissociation in the bovine alpha-crystallin [53]. Our study and earlier studies showed that the core of the beta-sheeted structures in alpha-crystallin at pH 7.5 remained intact under thermal denaturation temperatures up to 94 °C [28,32,34,35]. However, thermal stress above 50 °C induced a random-coil-like structure at pH 7.5.

Large conformational changes in alpha-crystallin occurred over the pH range of 1.0 to 7.5 (Figure 1). Initially, a loss of ellipticity at 217 nm occurred after shifting the pH from 7.5 to 5.0. Further reduction of the pH resulted in the formation of a beta-sheeted core structure in alpha-crystallin. Interestingly, the temperature did not affect the beta-sheeted secondary structure at all the pH values (7.5, 4.0, 2.0, and 1.0) tested; a slight gain of a beta-sheeted structure occurred at pH 4.0 and 1.0 (Figure 4). The formation of a random-coiled structure in alpha-crystallin occurred under two conditions: (i) >50 °C at pH 7.5 and (ii) 20 °C at pH 2.0.

Camel lens alpha-crystallin at pH undergoes a single thermal transition between 50 °C and 80 °C. After the thermal transition, whether alpha-crystallin was fully folded, partially folded, or fully denatured was unclear. However, the folding species was reversible. In the present study, the mid-point of the thermal transition (*T*_m_) was 60.9 ± 0.1 °C, and the enthalpy of denaturation was 237.0 ± 1.9 kJ/mol. In earlier studies, the thermal stability and structural changes of eye lens alpha-crystallin from other sources were determined [28,35,54]. Alpha-crystallin underwent a single thermal transition (*T*_m_) at approximately 61–64 °C [27,28,29]. The minor ambiguity in the *T*_m_ may be due to the difference in the detection methods (far-UV CD, DSC, or FTIR), buffer pH, ionic strength, or experimental conditions. The enthalpy of bovine lens alpha-crystallin was 235 kJ/mol by DSC [32]. Usually, the unfolding enthalpy of monomeric globular proteins of similar sizes is approximately 2.92 kJ/mol per residue [55]. Therefore, the calculated unfolding enthalpy of the alpha-crystallin subunits was more than 500 kJ/mol. Moreover, oligomerization increased the unfolding enthalpy of the proteins [56,57]. Although the crystal or NMR structure of alpha-crystallin is unavailable, data from multi-technique investigations have led to a consensus that subunit assembly of alpha-crystallin is controlled by the hydrophobicity of the N-terminal domains [50,51,58]. Therefore, dissociation of the oligomeric structure would result in the exposure of the buried hydrophobic patches, leading to an endothermic effect, which increases the overall unfolding enthalpy. Therefore, the calculated unfolding enthalpy of oligomeric alpha-crystallin at pH 7.5 may be much higher than 500 kJ/mol. Less than half of the enthalpic changes were determined in this study and earlier studies [32]. Accordingly, the DMS data presented in this work and an earlier study revealed that alpha-crystallin retained a secondary structure above the thermal transition (*T*_m_). These data also indicate that the thermal transition of alpha-crystallin does not dissociate its subunits. Denaturation without subunit dissociation was also observed in the Arc repressor [59]. The presence of a secondary structure in the alpha-crystallin or Arc repressor enabled a persistent interaction among subunits, maintaining the oligomeric structure during thermal stress.

Alpha-crystallin retained a native-like beta-sheeted secondary structure under thermal stress at pH 7.5, 4.0, 2.0, and 1.0, respectively. The unfolding enthalpies of alpha-crystallin at acidic pH values (4.0, 2.0, and 1.0, respectively) were less marked compared with those at pH 7.5 (Table 1). Thus, camel alpha-crystallin cannot be fully denatured at high temperatures (94 °C) and low pH values (e.g., pH 1.0).

Camel lens alpha-crystallin loses tertiary structure below pH 4.0 at room temperature (Figure 2). The tryptophan fluorescence spectra exhibited a 16 nm redshift at pH 2.0 and 20 °C. AlphaA-crystallin contains one tryptophan residue, whereas alphaB-crystallin contains two tryptophan residues [7]. These tryptophan residues are partially buried and located at the N-terminal domains (Supplementary Figure S2). Acid denaturation at pH 2.0 leads to partial unfolding of the N-terminal domain of alpha-crystallin. The complete unfolding of alpha-crystallin by chemical denaturants (6 M GdnHCl) resulted in a 27 nm redshift in tryptophan fluorescence (Figure 2). The far-UV CD data showed that camel alpha-crystallin retained a native-like beta-sheeted secondary structure at pH 2.0 but lost tertiary structure at this pH. Thus, alpha-crystallin forms a molten-like, globular structure at pH 2.0. Changes in the tertiary structure below pH 4.0 caused an increase in hydrophobicity (Figure 3). An increase in protonation below pH 2.0 caused charge–charge repulsion and forced alpha-crystallin to attain native-like secondary and tertiary structures (Figure 1 and Figure 2). Moreover, the unfolding enthalpy at pH 1.0 was closer to that of alpha-crystallin at pH 7.5 (Table 1).

Alpha-crystallin at pH 7.5 exhibited a slight increase in hydrophobicity above 35 °C and a large exposure of hydrophobic patches above 55 °C. Alpha-crystallin at acidic pH values (4.0, 2.0, and 1.0, respectively) resulted in little exposure of hydrophobic patches above 65 °C (Figure 6). We recently reported that the chaperoning effect of alpha-crystallin was activated in a stepwise manner and correlated with the biphasic changes in the tertiary structure and surface hydrophobicity of alpha-crystallin during thermal stress [7]. Alpha-crystallin even retained its chaperone activity above the post-transition temperature (89 °C) at pH 7.5 [7]. These data suggest that above the thermal transition state (denatured state), alpha-crystallin is not denatured. It retains a core beta-sheeted structure, maintains an oligomeric state, and performs chaperone activity efficiently (~90% protection at 89 °C when the alpha-crystallin to substrate *w*/*w* ratio was 0.87:1) [7]. This state is possible only when each monomeric subunit of alpha-crystallin retains a substrate-binding site (i.e., remains in the functional state). Therefore, a higher temperature does not denature alpha-crystallin at pH 7.5 but results in conformational changes required to activate its chaperone activities.

## 5. Conclusions

In this study, the thermodynamic parameters obtained during the thermal transition of camel lens alpha-crystallin and those determined in earlier studies for other alpha-crystallins represent less than half of the theoretically calculated values for complete protein denaturation. Even at acidic pH values, the enthalpies were much lower than those at pH 7.5. This finding showed that alpha-crystallin was never completely denatured at an extreme pH or temperature or both. Folding species formed post-transition were neither dissociated nor aggregated and were reversible. Interestingly, the folding species formed post-transition at pH 7.5 remained fully active (i.e., they retained almost a 1:1 substrate binding site). Maintaining a functional state post-thermal transition is a feature that is incongruent with the unfolded state. This phenomenon may be due to the natural selection of alpha-crystallin to suppress aggregation in the lens under stress and maintain clarity in the lens throughout life. These tasks may be more challenging to perform in the camel eye lens, which is exposed to high temperatures, solar radiation, and dryness from the desert climate. To overcome these larger tasks, camel lens alpha-crystallin has the natural ability to retain secondary and oligomeric structures and maintain solubility and activity at extreme temperatures.

## Figures and Tables

**Table 1:** Thermal transition midpoints ™ and enthalpies of alpha-crystallin at pH 1.0, 2.0, 4.0, and 7.5, respectively.

pH	Van’t Hoff Enthalpy (kJ/mol)	Transition Temperature (°C)
7.5	237.0 ± 1.9	60.9 ± 0.1
4.0	108.9 ± 4.7	48.1 ± 0.4
2.0	177.3 ± 9.2	59.1 ± 0.4
1.0	211.3 ± 10.2	43.5 ± 0.3

## Data Availability

Not applicable.

## Supplementary Materials

The following supporting information can be downloaded at: https://www.mdpi.com/article/10.3390/gels8050273/s1, Figure S1. Changes in far-UV CD signal at 217 nm at different pH values are plotted. With a de-crease in pH, the far-UV CD ellipticities initially decreased between pH 7.5 and 5.0. The far-UV CD ellipticities at 217 nm rapidly increased to a native-like structure below pH 5.0; Figure S2. Modeled 3D structure of camel lens alpha-crystallin. The tryptophan residue in the A- and B-chain of alpha-crystallin is shown in blue: (A) alphaA-crystallin and (B) alphaB-crystallin.
