# Supplementary material for: Modulation of the Structure and Stability of Novel Camel Lens Alpha-Crystallin by pH and Thermal Stress
Source: Gels. 2022 Apr 27;8(5):273. doi: 10.3390/gels8050273 (PMC9140948; doi:10.3390/gels8050273)
Supplement: Supplementary file 1 [file gels-08-00273-s001.zip › gels-1657946-supplementary.pdf]

gels

Supplementary Materials

Modulation of the Structure and Stability of Novel Camel Lens Alpha-Crystallin by pH and Thermal Stress

Ajamaluddin Malik [1,*], Javed Masood Khan [2], Abdullah S. Alhomida [1], Mohammad Shamsul Ola [1]

[1] Department of Biochemistry, College of Science, King Saud University, Riyadh 11451, Saudi Arabia; alhomida@ksu.edu.sa (A.S.A.); mola@ksu.edu.sa (M.S.O.)
[2] Department of Food Science and Nutrition, Faculty of Food and Agricultural Sciences, King Saud University, Riyadh 11451, Saudi Arabia; jmkhan@ksu.edu.sa
* Correspondence: amalik@ksu.edu.sa

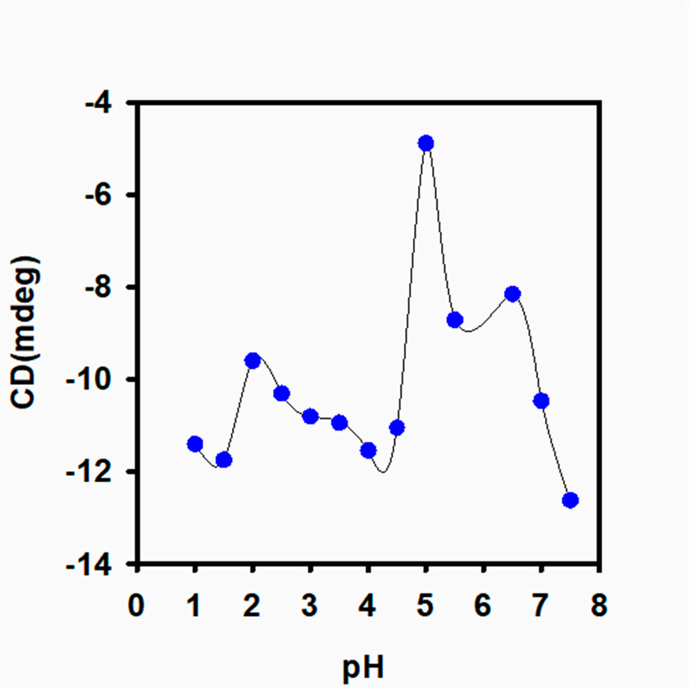

Figure S1. Changes in far-UV CD signal at 217 nm at different pH values are plotted. With a decrease in pH, the far-UV CD ellipticities initially decreased between pH 7.5 and 5.0. The far-UV CD ellipticities at 217 nm rapidly increased to a native-like structure below pH 5.0.

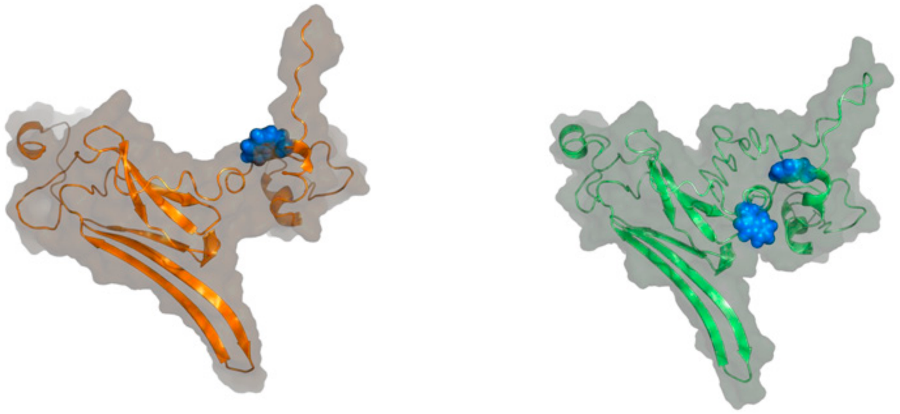

(**A**) (**B**)

Figure S2. Modeled 3D structure of camel lens alpha-crystallin. The tryptophan residue in the A- and B-chain of alpha-crystallin is shown in blue: (**A**) alphaA-crystallin and (**B**) alphaB-crystallin.
